# Daytime pharmacodynamic and pharmacokinetic evaluation of low-dose sublingual transmucosal zolpidem hemitartrate

**DOI:** 10.1002/hup.884

**Published:** 2008-01

**Authors:** Thomas Roth, David Mayleben, Bruce C Corser, Nikhilesh N Singh

**Affiliations:** 1 Henry Ford Hospital, Sleep Disorders and Research CenterDetroit, MI, USA; 2 Community ResearchCincinnati, OH, USA; 3 Sleep Management InstituteCincinnati, OH, USA; 4 Transcept Pharmaceuticals, Inc., Pt.Richmond, CA, USA

**Keywords:** zolpidem, pharmacodynamics, pharmacokinetics, MOTN insomnia

## Abstract

**Objectives:**

Buffered low-dose sublingual transmucosal zolpidem lozenge hemitartrate (ST zolpidem) is being developed for the treatment of middle-of-the-night insomnia. The objective of this double-blind placebo-controlled cross-over study (*n* = 24) was to evaluate the pharmacokinetics (PK) and daytime-sedative profile of 1.0, 1.75, and 3.5 mg dose of the formulation.

**Methods:**

Daytime sedation was measured pre-dose and up to 5 h post-dose objectively by the Digit Symbol Substitution Test (DSST) and subjectively using the Visual Analog Scale (VAS). Blood samples for PK assessment was collected pre-dose and up to 12 h post-dose.

**Results:**

The 1.75 and 3.5 mg, but not the 1 mg, ST zolpidem produced significant sedation versus placebo within 20 min of dosing which lasted for up to 3 h. Zolpidem from the formulation was rapidly absorbed and reached maximum plasma concentrations within 38 min of dosing, however the half-life was independent of the dose and side effects were consistent with the known pharmacology of the drug.

**Conclusions:**

ST zolpidem produced rapid, short duration of sedation and the effect was consistent with its PK profile. This novel low-dose formulation of zolpidem may provide clinicians and patients with a *prn* option for the management of sleep maintenance insomnia. Copyright © 2007 John Wiley & Sons, Ltd.

## INTRODUCTION

Zolpidem is extensively prescribed as a hypnotic in clinical practice, both in the form of a standard oral tablet (Ambien®) and a controlled-release tablet (Rosenberg, 2006; Soubrane *et al*., 2005). In either case, it is to be ingested at bedtime with an available 7–8 h of time in bed because of its potential for next-morning residual effects on memory and psychomotor performance following shorter periods of bedtime (Partinen *et al*., 2003; Verster *et al*., 2002). A different, low-dose sublingual transmucosal formulation of zolpidem (ST zolpidem, *Intermezzo*™) is currently under investigation as a sedative-hypnotic with rapid onset and short duration of action for the treatment of insomnia patients whose primary complaint is difficulty returning to sleep after middle-of-the-night awakening (Roth *et al*., 2006). This formulation consists of sublingual lozenges designed for transmucosal delivery of zolpidem.

The purpose of this study was to evaluate, in healthy volunteers after daytime administration, the pharmacodynamic (PD) and pharmacokinetic (PK) profiles and tolerability of ST zolpidem lozenges compared to placebo.

## METHODS

### Study design

This study was a single-dose, randomized, doubleblind, placebo-controlled, daytime, cross-over study. Three doses of ST zolpidem (1.0, 1.75, and 3.5 mg) were compared with matching placebo in healthy volunteers. The protocol for this study was approved by an institutional review board for the study site and the study itself was conducted in accordance with the Declaration of Helsinki and the Good Clinical Practice guidelines. Subjects were paid for their participation.

Subject selection included a clinical assessment visit and 7 days of morning sleep diary screening to ensure that all study criteria were met. Subjects were randomized to one of four treatment sequences, which included all three doses of active treatment and placebo. Each treatment period consisted of 2 days separated by a washout period of 5–12 days.

During each of the four treatment periods, subjects were admitted to the site on the evening prior to dosing and had an obligatory 8 h in bed. The following two mornings, subjects were awakened at a fixed time and, following baseline assessments, received the study drug at 8:00 AM (approximately 1 h after awakening). PD assessments were conducted prior to dosing and over a period of 5 h after study drug administration on the first morning of treatment. On the second morning, the same treatment was administered and venous blood was drawn prior to dosing and over a period of 12 h following study treatment administration for PK evaluation.

In each treatment period, subject mobility was limited. Specifically, for the first 5 h after dose administration, participants were required to remain seated unless medically or procedurally necessary. Furthermore, subjects were kept awake until all procedures were completed. Subjects had to pass a heel-to-toe gait test prior to leaving the laboratory.

### Subject recruitment and selection

Healthy, non-smoking adult men and women, aged 21–44 with no current self-reported sleeping problems were eligible for participation in the study. After signing a written informed consent statement and following initial screening, a physical examination, clinical laboratory tests, and electrocardiogram, subjects were invited to complete a 7-day sleep diary provided that they did not (1) have any DSM-IV Axis I psychiatric disorders or any circadian rhythm sleep disorder, (2) have a history of substance abuse or substance dependence, (3) have a Epworth Sleepiness Scale score of greater than 12, (4) have had an acute clinically significant illness or surgery, including oral surgery, tooth extraction, or piercing of the lip/tongue within 60 days prior to Day 1 of the study, (5) utilize any over-the-counter or prescription medication within 2 weeks prior to screening, or (6) take any drugs known to induce or inhibit hepatic drug metabolism within 30 days prior to Day 1 of double-blind study medication.

Subjects qualified for randomization if their diaries reported a mean weekly latency to sleep onset of ≤30 min, a mean weekly total time in bed of ≥7 h, and a stable bedtime pattern as defined by a usual bedtime between 2200 and 2400 and a usual rise time between 0500 and 0800 (neither of which varied by more than 2 h on 5 of 7 nights).

### Study procedures

*Study drug*. The four treatments evaluated were 1.0, 1.75, and 3.5 mg ST zolpidem and placebo lozenges. Subjects were randomized into dosing sequences of four treatment periods (Latin Square) that were separated by 5–12 days. Each subject was randomized into a dosing sequence that included all four treatments. Medication was dispensed by study personnel on each morning in the sleep laboratory at 8 AM.

Subjects were instructed to rinse their mouth with water prior to dosing and then place the lozenge under their tongue until it dissolved. Saliva was swallowed every 2min until the nearest 2 min after complete lozenge dissolution. Study personnel performed oral cavity examinations before and after dosing to ensure consumption of medication and to note any signs of oral irritation.

*PD assessments*. Subjects practiced PD tests after admission to the laboratory on the night prior to treatment. On the first morning of each treatment period, subjects performed the PD tests immediately before dosing and at 10 min [visual analog scale (VAS) only], 20 min, 1, 1.5, 2, 2.5, 3, 4, and 5 h post-dose. PD tests were always performed in the same order: Digit Symbol Substitution Test (DSST), Choice Reaction Test (CRT), Symbol Copying Test (SCT), subject rating of sedation (VAS) and Word Recall Test.

During the DSST (Kaplan *et al*., 1997), subjects were given a set of symbols with corresponding single digit numbers and a set of ‘blank’ boxes with corresponding digits. Subjects were asked to make as many symbol-for-digit substitutions as possible working from left to right without skipping any boxes within a 90-s period and the number of correct substitutions was recorded. Throughout the study, subjects completed equivalent DSST variants, with no individual taking the same form more than once.

For the CRT (Roehrs *et al*., 1994), subjects were provided with a hand-held device with response buttons for measuring reaction time following the presentation of visual and/or audio stimulus. Response time was defined as the time in milliseconds between the onset of the stimulus and the response button being pressed. The mean response time, the number of errors, and the number of lapses (defined as reaction time >500 ms) were evaluated.

During the SCT (Stone, 1984), subjects were given a sheet filled with double rows: the upper row filled with symbols, the lower row empty. Subjects were asked to make as many accurate symbol-copies as possible working from left to right without skipping any boxes within a 90-s period and the number of correct copies was recorded. Throughout the study, subjects completed equivalent SCT variants, with no individual taking the same form more than once.

Finally, acquisition and immediate recall of information was evaluated using a word-list free recall procedure (Shader *et al*., 1986). Fifteen words were read in random order at a rate of one word per second, during each test session. Recall was tested immediately after presentation of the list, and subjects were given 1 min to write down list items recalled in any order. Throughout the study, subjects had to recall equivalent word-list variants, with no individual hearing the same list more than once. The number of correct words (ignoring spelling mistakes) was recorded.

*Subjective ratings*. Subjects' self-ratings of sedative effects were obtained on a 100mm VAS anchored by ‘0’ = ‘very sleepy’ and 100' = ‘wide awake and alert’. This type of VAS scale is often used in clinical trials to assay sedative effects (typically as residual effects in the morning).

*PK sample collection and parameters*. On the second morning of the treatment period, a total of 18 blood samples were collected. The first sample was collected prior to dosing. Subsequent samples were collected at 5, 10, 20, 30, and 45 min and 1, 1.5, 2, 2.5, 3, 3.5, 4, 5, 6, 8, 10, and 12 h post-dose. All blood samples were centrifuged within 10 min and plasma was separated, divided into two duplicate aliquots, and frozen until the time of assay. The bioanalytical laboratory analyzed zolpidem in plasma samples using a validated LC/ MS/MS method. PK parameters included the area under the plasma concentration curve from time 0 to the last measurable concentration (AUC_0-*t*_), the area under the plasma concentration curve from time 0 to infinity (AUC_0-inf_), the maximum plasma concentration (*C*_max_), the time of the maximum plasma concentration (*t*_max_), and the apparent terminal elimination half-life (*t*_1/2_).

### Safety evaluations

Vital signs were recorded at screening, prior to dosing and at scheduled intervals during each treatment period. Subjects oral cavities were examined for buccal irritation prior to dosing, at the time of lozenge dissolution, at 15, 30, 60, and 120 min post-dissolution and at discharge. A physical examination along with chemistry, hematology, and urinalysis were performed at study entry and prior to discharge in the fourth treatment period. All subjects had to pass a heel-to-toe gait test before leaving the clinic.

### Statistical analysis

All analyses performed in this study were defined prior to breaking the study blind. All randomized subjects completed all four treatment periods. Therefore, the intent-to-treat and per-protocol populations were identical. The statistical analyses discussed reflect the full set of 24 randomized patients.

PD values are presented and analyzed as change relative to pre-dose values. Each time point was evaluated separately relative to the baseline value. In addition, area under the time-effect curve for the effect change scores was calculated for defined time intervals.

PK parameters were calculated from the concentration–time data using non-compartmental techniques. Using SAS, ANOVA was performed on untransformed *t*_max_ and *t*_1/2_, and on ln-transformed dose normalized values of AUC_0-*t*_, AUC_0-inf_, and *C*_max_ at the alpha level of 0.05. Linearity in PK response of various doses was assessed by applying the power function *P* = A^*^Dose^*b*^ to non-normalized *C*_max_ and AUC_0-*t*_ values of zolpidem.

Safety was assessed by adverse events (AEs), vital signs, and laboratory parameters. AEs were defined according to the Medical Dictionary for Regulatory Activities (MedDRA®). AEs with onset (or worsening) after the start of study drug were considered treatment-emergent. The frequency of treatmentemergent AEs and the frequency of events by body system were summarized by treatment period according to preferred term and system organ class.

## RESULTS

### Demographics

A total of 24 subjects were randomized to treatment for this study. All participants completed all four treatment periods; there were no discontinuations. The demographics and sleep histories of the subject population are detailed in Tables 1 and 2. As can be seen, study subjects were healthy and reported no sleep difficulties.

**Table 1:** Subject demographics

Gender	
Male (%)	13 (54.2)
Female (%)	11 (45.8)
Race	
Caucasian (%)	15 (62.5)
African-American (%)	9 (32.5)
Age	
Mean (SD)	34.7 (7.1)
Range	21–44
Weight (kg)	
Mean (SD)	74.4 (10.8)
Range	51.7–100.2
BMI	
Mean (SD)	24.9 (2.8)
Range	19–30

**Table 2:** Subject sleep history

Usual time in bed (h)	
Mean (SD)	8.2 (0.4)
Range	8.0–9.0
Usual time to fall asleep (min)	
Mean (SD)	13.0 (5.4)
Range	3.0–25.0
Usual sleep time during night (h)	
Mean (SD)	8.1 (0.4)
Range	7.5–9.0
Usual time awake during night (min)	
Mean (SD)	2.3 (2.8)
Range	0.0–10.0
Usual number of nocturnal awakenings	
0	13
1	10
2	1
Epworth sleepiness scale	
Mean (SD)	3.5 (2.6)
Range	0.0–11.0

### Psychomotor performance

The sedative effects of ST zolpidem lozenges were assessed by multiple PD evaluations, including DSST, CRT, SCT, and Word Recall as well as by subjective self-rating of sedation by VAS. On the pre-drug performance sessions, no significant treatment differences were observed on any of these endpoints. During post-drug performance, in comparison to placebo, all measures were significantly affected by at least one dose of ST zolpidem.

**Figure 1:**
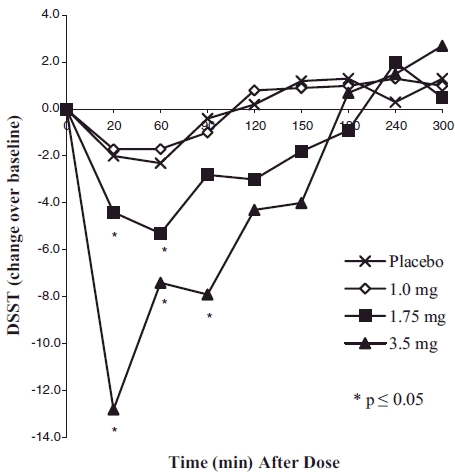
Mean change over baseline in DSST scores

**Figure 2:**
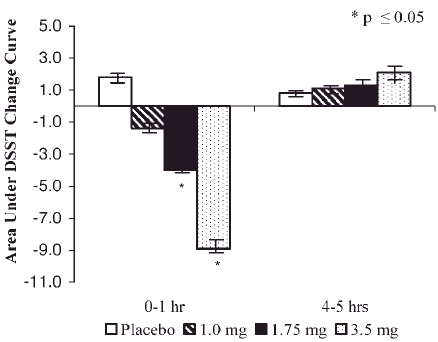
Mean±SEM 1 h effect areas for changes over baseline in DSST scores

DSST scores at individual time points indicated significant psychomotor impairment by ST zolpidem 3.5 and 1.75 mg as early as 20 min post-intake (Figure 1). Significant reduction in DSST scores lasted up to 90 min post-dose (3.5 mg), and performance after ST zolpidem was no longer distinguishable from placebo on any endpoint as early as at the 3 h time point. These observations were confirmed by partial 1 h effect-area measures (Figure 2). There was significant impairment compared to placebo for ST zolpidem 1.75 mg and 3.5 mg during the (0–1) h time period, while there was no longer any difference during the (4–5) h time period. ST zolpidem 1 mg had no measurable effect by either analysis.

Relevant characteristics of the other PD evaluations are summarized in Table 3. Overall, it is readily apparent that ST zolpidem at the 1mg dose has no measurable effect on any parameter (except at one time point measuring the number of errors in CRT), whereas ST zolpidem 3.5 mg impacts all outcome measures, albeit for different time periods. Based on these tests, the time of maximum impairment by ST zolpidem 1.75 and 3.5 mg ranges from 20 min to 2 h post-dose, and time post-drug where the measured parameters no longer differed from placebo after 2 h.

**Table 3:** Effect of ST zolpidem on daytime PD assessments

Parameter	ST zolpidem dosage (mg)	Maximum change relative to placebo	*p*-value	Time of maximum change	Time no longer different from placebo
Word Recall (# words)	3.5	1.2	0.0387	20 min	1 h
	1.75	1.0	N.S.	1 h, 2 h	N.A.
	1.0	0.6	N.S.	1 h	N.A.
CRT (reaction time, ms)	3.5	234.7	<0.0001	20 min	2 h
	1.75	103.3	N.S.	1 h	N.A.
	1.0	85.7	N.S.	1 h	N.A.
CRT (# lapses)	3.5	13.6	<0.0001	20 min	2.5 h
	1.75	5.6	0.0199	20 min	1 h
	1.0	4.3	N.S.	1 h	N.A.
CRT (# errors)	3.5	5.1	0.0225	3 h	4 h
	1.75	3.1	N.S.	2.5 h	N.A.
	1.0	6.8	0.0419	1 h	1.5 h
SCT	3.5	14.8	<0.0001	20 min	2.0 h
	1.75	7.6	0.0011	1 h	1.5 h
	1.0	3.0	N.S.	1 h	N.A.

Specifically, onset of impairment of CRT was found to be as early as the other PD outcomes, but duration was differentially affected depending on the specific parameter. Actual reaction time was significantly prolonged by zolpidem 3.5 mg at the early time points only and was no longer different from placebo at 2 h post-drug administration. The number of lapses was affected by both 3.5 and 1.75 mg ST zolpidem, with peak effect for both at 20 min, but duration of impairment was longer for the 3.5 mg than the 1.75 mg dose, 2.5 h, and 1.0 h, respectively. The number of errors committed during CRT measures was found to be somewhat variable. The 3.5 mg dose was associated with the longest duration of impairment with a peak effect at 3 h and subsequently, no statistical difference from placebo at 4 h. Although the 1.75 mg dose did not differ at any time point from placebo, there was one statistically significant increase in the number of errors after the 1mg dose, occurring at the 1 h time point (Table 3).

The two higher doses of ST zolpidem, that is, 3.5 and 1.75 mg, significantly impaired fine motor activity as measured by SCT, with impairment due to the higher dose lasting 30 min longer than the lower, 1.5 h versus 1 h, respectively (Table 3).

Lastly, in terms of memory, compared to placebo, immediate free recall was significantly impaired by ST zolpidem 3.5 mg at 20 min post-ingestion and this effect was no longer detectable 1 h later. No measurable effect was observed with the two lower doses of ST zolpidem (Table 3).

### Subjective ratings

**Figure 3:**
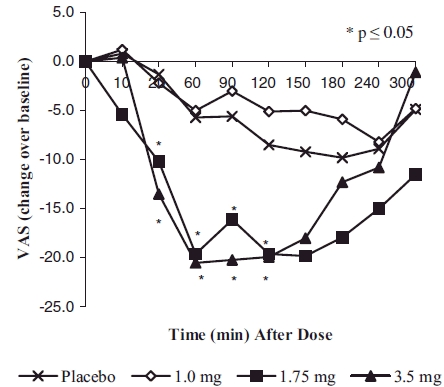
Mean change over baseline in scores of self-rated sedation on 100mm VAS

Self-ratings of sedation by the VAS exhibited a pattern similar to that observed for DSST (Figure 3). Subjects did not feel sedated at 10 min post-drug intake, but rated themselves significantly sedated compared to placebo from 20 min through 2 h post-drug at the 1.75 and 3.5 mg dose levels. The ratings remained different from placebo for up to 3 h, but were no longer statistically significantly different, primarily due to progressively increased sedation rating in the placebo condition.

### PK

**Figure 4:**
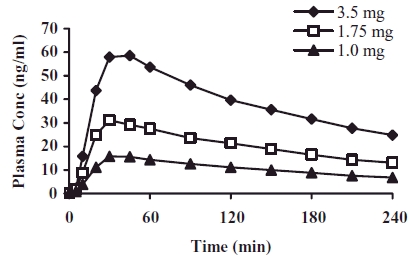
Plasma concentration–time profiles of zolpidem following ST zolpidem administration

Descriptive statistics for the PK parameters are presented by dose in Table 4. Over the dose range and time periods studied, mean *C*_max_ and mean AUC values were proportional to dose. Mean *t*_max_ and mean elimination half-life were equivalent across treatment conditions. Plasma concentration–time profiles following ST zolpidem administration are presented in Figure 4. Zolpidem plasma levels of >20–25 ng/ml were reached within 20 min after both 1.75 and 3.5 mg ST zolpidem administration and were maintained for up to 4 h. Zolpidem was no longer detectable 12 h after administration.

**Table 4:** Mean PK parameters (SD) of ST zolpidem

	1.0mg	1.75mg	3.5mg
*C*_max_ (ng/ml)	17.03 (6.84)	32.17 (10.38)	64.14 (22.36)
Range *C*_max_	0–35.51	9.33–60.33	19.85–125.96
*t*_1/2_ (h)	2.33 (0.79)	2.43 (0.60)	2.45 (0.58)
AUC_0-inf_ (ng*h/ml)	66.16 (31.49)	126.10 (53.39)	242.57 (100.37)
*t*_max_ (min)	35.7 (12.7)	37.9 (16.1)	37.9 (12.3)

### Safety

The ST zolpidem lozenges were generally safe and well tolerated. Subjects experienced a total of 48 AEs, most of which were related to the clinical effect of the drug sedation and were mild-to-moderate in severity (Table 5). Side effects appeared only at the high dose, with 10 subjects reporting sedation at 3.5 mg compared to 3 subjects for placebo. Dizziness, nausea, and headache peaked at the 3.5 mg dose level (three, three and two subjects, respectively), with fewer instances seen with the 1.75 mg dose (one, zero, and two subjects) and no reports of these conditions at either the 1.0 mg level or placebo. Only one event (epigastric pain) was severe and was judged unrelated to treatment (1.75 mg lozenge) by the investigator. Two AEs not related to treatment (headache: 1.75 mg lozenge, dysmenorrhoea: placebo) were treated with Tylenol or ibuprofen. All other events resolved without treatment.

**Table 5:** AEs occurring in ≥5% of subjects

Variable	Placebo	1.0 mg	1.75 mg	3.5mg
Somnolence	3 (12.5%)	5 (20.8%)	3 (12.5%)	10 (41.7%)
Fatigue	6 (25.0)	2 (8.3)	8 (33.3)	4 (16.7)
Dizziness	—	—	1 (4.2)	3 (12.5)
Nausea	—	—	—	3 (12.5)
Headache	—	—	1 (4.2)	2 (8.3)

## DISCUSSION

Middle-of-the-night awakening with difficulty returning to sleep is a common complaint in chronic insomnia patients (Shochat *et al*., 1999). According to the National Sleep Foundation's 1995 ‘Sleep in America’ poll, about 20% of the US population may be suffering from MOTN insomnia and the prevalence in primary care patients may be even higher (Hohagen *et al*., 1994a, 1994b; Mellinger *et al*., 1985). Many patients with this kind of insomnia do not experience MOTN awakenings every night, but may dose themselves prophylactically with a hypnotic each evening prior to sleep, since presently all approved sedative hypnotics are indicated for pre-sleep use only. Ideally, however, MOTN sleep disruptions should be managed by MOTN dosing, only when the symptoms occur. A sedative-hypnotic with rapid onset and short duration would be ideal for such treatment. Low-dose, transmucosal zolpidem (ST zolpidem) is being developed to provide clinicians and patients with such an option for the management of MOTN insomnia. The present study evaluated, in healthy volunteers, the PD/PK of single doses of 1.0, 1.75, and 3.5 mg ST zolpidem following daytime administration.

Specific PD outcome measures included DSST, Choice Reaction Time, SCT, Word Recall, and scoring on a self-rating 100mm VAS of sedation, all of which have been used extensively for the evaluation of the immediate performance-disruptive effects of sedativehypnotic drugs (Greenblatt *et al*., 1998) or their morning residual effects following their bedtime administration (Piergies *et al*., 1996; Scharf *et al*., 1994).

It is noteworthy that this study was conducted in normal sleepers with zolpidem intake early in the morning subsequent to a full night's sleep. Although in this study, no direct comparison was included with zolpidem 5 or 10 mg in standard oral formulations, published observations of very similar study design indicate that following the 10 mg zolpidem dose, measurable performance deficit occurs at 1 h postintake and is of similar magnitude as measured here for the 3.5 mg dose (Greenblatt *et al*., 1998). Thus, it appears that sedative effects of ST zolpidem occurred at a lower dose and at a time less than half of those reported for oral zolpidem 10 mg (Ambien® 10 mg). Within the ST zolpidemdose range investigated in this study (1–3.5 mg), there was a reasonable dose–effect relationship with 3.5 mg showing the greatest sedative potential and 1.75 mg as the lowest active dose. The sublingual dose of 1mg can be considered a no-effect ST zolpidem dose.

The PK profile of ST zolpidem lozenges is characterized by very rapid absorption with mean peak concentrations of 17.8 (range 0–35.5), 32.2 (range 9.3–60.3), and 64.1 (range 19.9–125.9) ng/ml for 1.0, 1.75, and 3.5 mg of ST zolpidem, respectively, occurring at approximately 37 (range 36–37.9) minutes post-administration. In comparison, currently available oral zolpidem tablets (Ambien®) are reported to attain peak concentrations (*C*_max_) of 59 (range 29–113) and 121 (range 58–272) ng/ml for 5 and 10 mg, respectively, at a mean time (*t*_max_) of 1.6 h for both (Ambien® Package Insert). Thus, *t*_max_ for ST zolpidem occurs at a time less than half of that reported of the oral zolpidem tablets.

In addition, within 20 min post-dose, ST zolpidem 1.75 and 3.5 mg achieved plasma zolpidem levels greater than 20 to 25 ng/ml, the estimated levels for onset and offset of sedation (Patat *et al*., 1994). These reportedly clinically relevant zolpidem blood levels are paralleled by the PD observations of sedative activity, specifically the effects on DSST scores and subjective ratings of sedation. ST zolpidem did not alter the elimination half-life of zolpidem: *t*_1/2_ of ST zolpidem (2.3, 2.4, and 2.5 h for 1, 1.75, and 3.5 mg, respectively) is very much in agreement with that reported for oral zolpidem tablets (2.5 and 2.6 h for 5 and 10 mg, respectively).

ST zolpidem lozenges were found to be generally safe and well tolerated. The side effect profile was consistent with the low-dose sedative-hypnotic effects of zolpidem.

Taken together, these results suggest that ST zolpidem 3.5 mg produced sedative activity similar to the sedative effects reported for 10 mg oral zolpidem. Furthermore, the maximal sedative effect as measured by DSST produced peak by ST zolpidem was observed as early as 20 min post-dose as compared to 60 min post-dose reported for 10 mg oral zolpidem (Greenblatt *et al*., 1998). These PD effects of ST zolpidem may be related to its PK as suggested by a shorter *t*_max_ for ST zolpidem than that reported for 10 mg oral zolpidem. Lastly, ST zolpidem produced rapid clinically relevant blood levels which persisted for 2–4 h which were paralleled with PD assays sedative activity. It may be concluded that these characteristics make ST zolpidem an ideal candidate for the *prn* treatment of sleep maintenance insomnia characterized by prolonged wakefulness after middle-of-the-night awakenings.
